# 3D magnetic field modeling and beam optics validation for a static gantry system

**DOI:** 10.1007/s12194-026-01120-3

**Published:** 2026-08-31

**Authors:** H. Zhao, M. Fukuda, A. Gerbershagen, H. Kanda, K. P. Nesteruk, J. M. Schippers

**Affiliations:** 1 Research Center for Nuclear Physics (RCNP), The University of Osaka, 10-1 Mihogaaka, Ibaraki, Osaka 567-0047 Japan; 2 Particle Therapy Research Center (PARTREC), University Medical Center Groningen, University of Groningen, Zernikelaan 25, Groningen, 9747 AA The Netherlands; 3 Department of Radiation Oncology, Mass General Brigham Cancer Institute and Harvard Medical School, 55 Fruit Street, Boston, MA 02114 USA; 4 Paul Scherrer Institute (PSI), Forschungsstrasse 111, Villigen, 5232 Switzerland

**Keywords:** Proton arc therapy, Static gantry, 3D magnetic field modeling, Beam optics, Edge focusing

## Abstract

In this work, we developed a 3D computational framework to evaluate the beam transport performance of a static gantry proposed in previous studies. This static gantry enables magnetically driven azimuthal angle selection via tailored fields rather than mechanical rotation, yet its practical feasibility requires rigorous simulation-based evaluation. Our approach utilizes full 3D magnetic field modeling in Opera3D coupled with a custom phase-ellipse tracking program. This integrated workflow captures realistic 3D fringe-field effects and quantitatively evaluates the optical limits driven by momentum spread and boundary edge-focusing. By identifying these primary limitations, this platform provides the requirements and a practical foundation to guide the development and optimization of future static gantry designs.

## Introduction

While advanced delivery methods like proton arc therapy (PAT) improve dose conformity and reduce normal tissue toxicity [1–3], their clinical implementation is hindered by the mechanical latency and efficiency constraints of standard rotating gantries [4–7]. To mitigate this, a static gantry concept was proposed, performing rapid azimuthal angle modulation via dynamic magnetic field control and an upstream Injection Scanning Magnet (ISM) [8].

Although initial studies established its conceptual design using idealized 2D analytical extrapolations [8], transitioning toward a robust beam optic design requires 3D numerical evaluations. Analytical approximations are inherently insufficient for resolving complex fringe fields and ferromagnetic saturation in compact 3D spaces.

*Scope of this work.* We developed a 3D computational framework integrating Opera3D magnetic modeling [9] with custom phase-space tracking to evaluate the static gantry. Rather than proposing a finalized clinical design, we identify the primary optical limitations within an unoptimized baseline field. Analyzing these transport characteristics provides a practical foundation for future 3D field optimization.

## Methods

### System concept and structural configuration

**Fig. 1:**
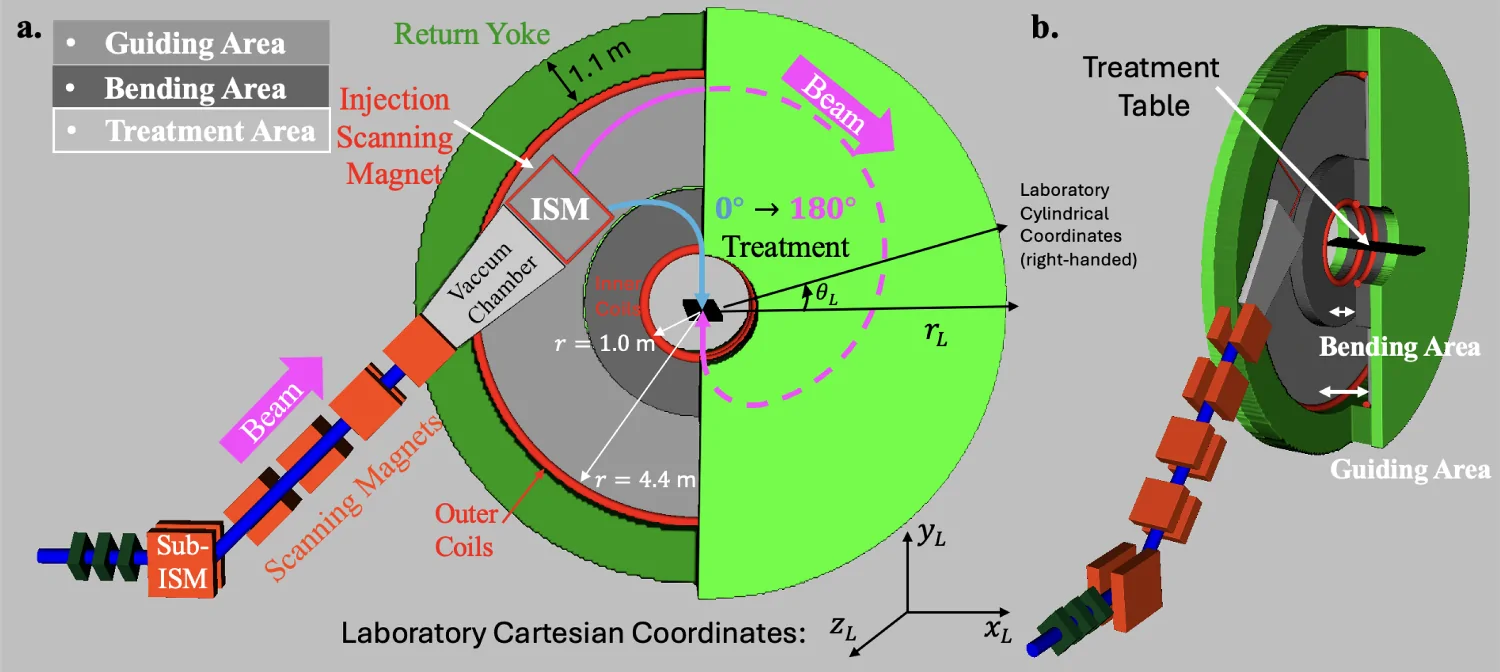
**a** Front view of the static gantry structure. Magnet poles for the guiding area and the bending area are illustrated in light gray and dark gray, respectively. **b** Side view of the structure showing the area-dependent pole gaps

To facilitate the 3D magnetic field modeling in Opera3D, we established a quantitative structural layout based on the conceptual design from [8], defining specific dimensions such as the return yoke thickness and area-dependent pole gaps. As illustrated in Fig. 1, the static gantry surrounds the patient with a cylindrical magnetic field. The primary magnetic flux is excited by a set of outer coils. An auxiliary set of inner coils actively cancels the field to maintain a safe treatment area. For a 230 MeV proton beam, the magnetic field is estimated to exceed 2 T for this compact system. To achieve such high magnetic fields, we plan to utilize High-Temperature Superconducting (HTS) coils. To keep more freedom for parameters in magnetic field optimization, the total device radius is increased to 5.5 m. This dimension explicitly includes a 1.1 m thick return yoke to safely contain the internal magnetic flux. The internal magnetic volume is divided radially into three functional areas using laboratory cylindrical coordinates $(r_L,\theta _L,z_L)$. A guiding area ($r_L=2.2$ to $4.4~\textrm{m}$) utilizes a pole gap of $94~\textrm{cm}$. A bending area ($r_L=1.0$ to $2.2~\textrm{m}$) uses a narrower $30~\textrm{cm}$ gap to establish the required higher local fields. Finally, the treatment area ($r_L<1.0~\textrm{m}$) surrounds the patient.

The therapeutic beam enters through an Injection Scanning Magnet (ISM) located at a fixed azimuth of $\theta _L=135^\circ$. At the current stage of development, we focus exclusively on the core 3D magnetic field design and the internal beam optics. The detailed engineering design of the injection beamline, including the ISM, and necessary clinical instrumentation will be addressed once these internal optical challenges are resolved.

### Magnetic field modeling and data extraction

We constructed the magnetic field model in Opera3D based on the structural configuration described in Sect. 2.1. The injection beamline hardware is explicitly omitted at this stage. Cartesian field components $(B_x,B_y,B_z)$ are exported from this model on polar patch rasters at four positive axial slices $z_L=\{0,\,0.5,\,1.0,\,1.5\}\,\textrm{cm}$. The extracted domain covers $r_L\in [0,600]~\textrm{cm}$ and $\theta _L\in [-180^\circ ,180^\circ ]$ with a typical sampling resolution of $\Delta r_L=5~\textrm{mm}$ and $\Delta \theta _L=0.5^\circ$. These outputs are post-processed into cylindrical components $(B_r,B_\theta ,B_z)$ to perform the beam tracking. For the region where $z_L < 0$, the magnetic field is derived explicitly by applying mid-plane symmetry, where the transverse components $B_r$ and $B_\theta$ reverse their signs while the axial component $B_z$ remains symmetric. During the 3D transport evaluations, continuous magnetic field values between the discrete grid points are queried utilizing trilinear interpolation.

### Reference trajectory backtracking

To calculate reference trajectories for each treatment angle $\theta _T\in \{0^\circ ,1^\circ ,\dots ,180^\circ \}$, a single $230~\textrm{MeV}$ proton ($\beta \approx 0.596$) is backtracked from isocenter along the outward radial direction $\textbf{e}_r(\theta _T)$ on the mid-plane $z_L=0$.

The particle trajectories were backtracked by integrating the Lorentz force equation using a custom 4th-order Runge–Kutta (RK4) numerical integrator. To ensure high computational fidelity, we employed a sufficiently small time step of $\Delta t=10^{-12}~\textrm{s}$ (corresponding to a spatial step length of $\Delta s \approx 0.18~\textrm{mm}$). Convergence tests demonstrated that further halving the time step to $0.5 \times 10^{-12}~\textrm{s}$ resulted in a target position shift of less than $0.005~\textrm{mm}$. During integration, the magnetic field at each particle’s position is queried via trilinear interpolation of the 3D maps described in Sect. 2.2.

The position $(r_L,\theta _L)$ is continuously updated until the ISM exit plane is intersected. This intersection defines the *injection condition* $(r_0,\ \varphi _0)$ as follows:

1 $$\begin{aligned} r_0 \equiv r_L(\textbf{x}_{\textrm{exit}}) \end{aligned}$$

2 $$\begin{aligned} \varphi _0 \equiv \arcsin \!\left( \frac{[\textbf{v}(\textbf{x}_{\textrm{exit}})\times \textbf{n}_{\textrm{ISM}}]\cdot \textbf{e}_z}{\Vert \textbf{v}(\textbf{x}_{\textrm{exit}})\Vert }\right) \end{aligned}$$

where $\textbf{n}_{\textrm{ISM}}$ is the outward normal of the exit face in the mid-plane, and $\textbf{e}_z$ is the laboratory axial unit vector. This signed definition ensures consistency with the negative entrance angles required for specific treatment orientations.

### Rationale and approach

To resolve *how* specific initial phase space parameters (Twiss parameters) are transported in this concept, a custom *ellipse-edge sampling* strategy (abbrev. EES) is adopted, and each sampled particle is tracked independently through the 3D field maps.

#### Initial condition sampling

***Phase-space sampling***

**Fig. 2:**
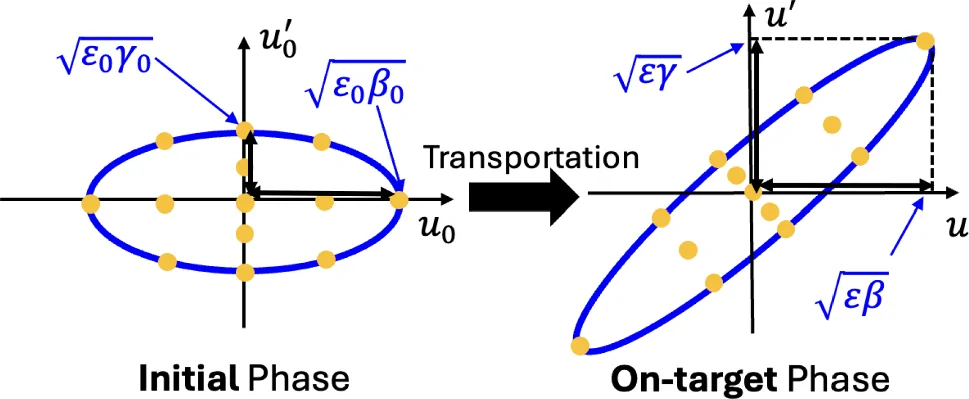
Sampling of the selected 13-point on the phase space ellipse at the ISM exit (left) and its schematic transport (right). Emittance is preserved; orientation/aspect vary with optics

General-purpose beam-dynamics codes can propagate particle ensembles. Here we used a compact deterministic sampler to evaluate the beam transport more efficiently in this particular case. At the ISM exit, we assume an upright phase ellipse representing a beam waist ($\alpha _0=0$) for each transverse plane. As illustrated in Fig. 2, we probe this space using a symmetric 13-point set that includes the origin, maximum intercepts, half-maximum intercepts, and intermediate boundary points. To couple the horizontal and vertical planes consistently, we impose a specific real-space correspondence rule. Whenever one plane assumes a spatial offset, the orthogonal plane maintains a zero spatial offset and scans only angular slopes. This systematic combination builds a comprehensive set of 105 transverse particles per treatment angle.

***Momentum spread sampling and forward tracking***

To systematically evaluate chromatic dispersion, we define $\delta \equiv \Delta p/p_0$ and sample momentum deviations containing *0*, $\pm 0.5\delta$, and $\pm \delta$. This yields *5 × 105* distinct tracked particles per treatment angle.

To evaluate the beam envelope, these 525 particles are then tracked forward from the ISM exit to the target. This forward propagation utilizes the exact same RK4 numerical integrator and time step described in Sect. 2.3, differing only in the starting location and the integration termination condition at the isocenter.

#### Initial beam condition at ISM exit

**Table 1:** Assumed initial beam parameters for 230 MeV proton at the ISM exit used for transport evaluation (applied to both *x* and *y* planes)

Parameter	Value
$\varepsilon _{0}$ (geometric)	1.0 ×10^-6^ m ⋅ rad
*β*_0_	9.0 m
*α*_0_	0
$\gamma _{0}=(1+\alpha _0^2)/\beta _0$	0.11 m^-1^
$(\Delta p/p_{0})_{0}$	± 1.0% and ± 0.10%

To prevent premature beam loss due to clipping against the pole gap within the unoptimized baseline magnetic field, we assume a conservative initial phase space. This approach cleanly isolates the intrinsic optical magnification and the chromatic dispersion driven by the magnetic field. Therefore, we assume identical transverse Twiss parameters in both planes. Furthermore, to explicitly differentiate the effects of initial Twiss parameters and momentum spread in the two transverse planes, we evaluate the transport under two distinct momentum spread conditions ($\pm 1.0\%$ and $\pm 0.1\%$) at the ISM exit. The values are listed in Table 1. The corresponding rms envelope at the exit is thus:

$$ \sigma _{u,0}= 3.0\ \textrm{mm},\qquad \sigma '_{u,0}= 0.33\ \textrm{mrad}. $$

## Results

### 3D magnetic field profile

**Fig. 3:**
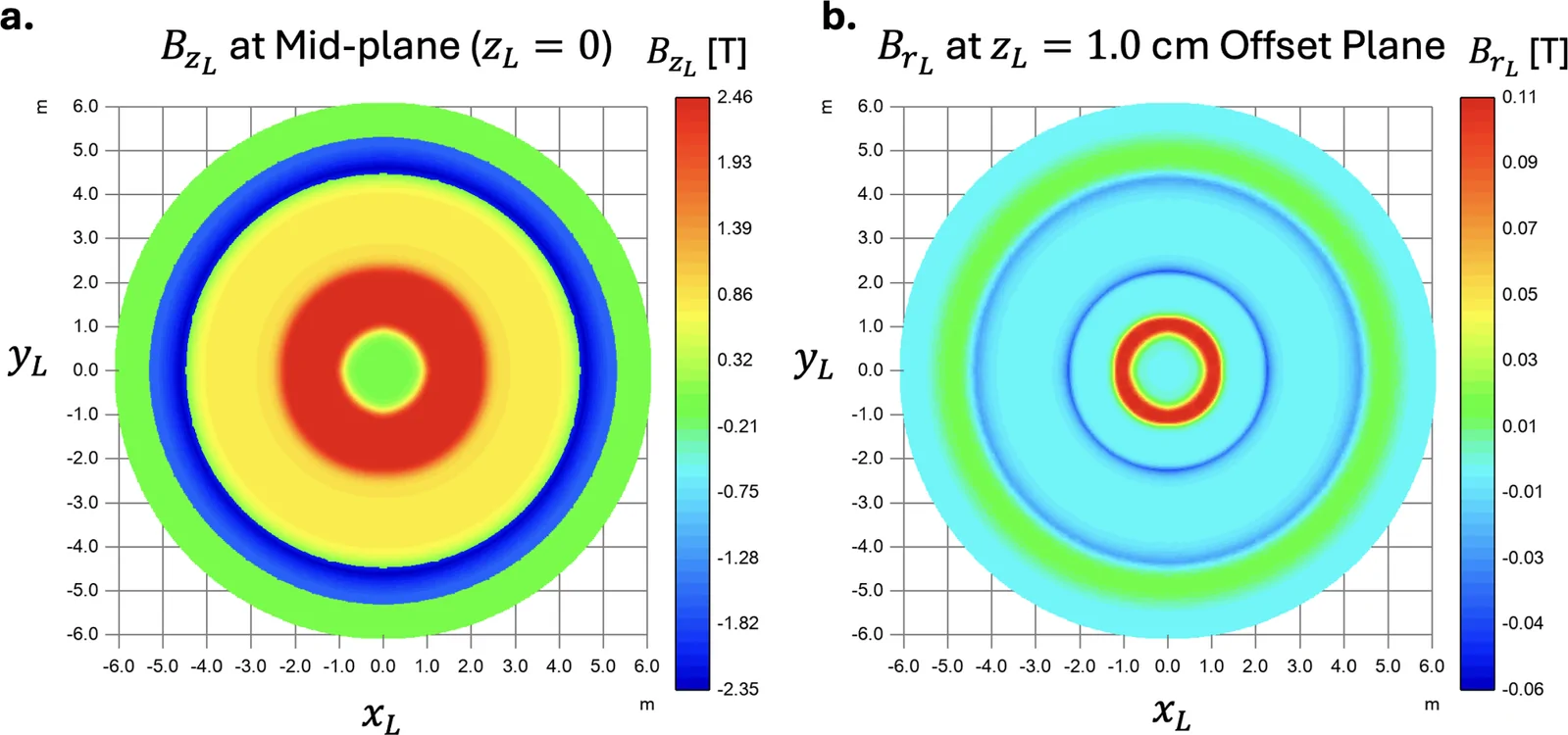
Magnetic field components replotted with Graph-R. **a** The axial magnetic field $B_z(r_L,\theta _L,0)$ on the mid-plane. **b** The radial component $B_r$ evaluated on an offset plane at $z_L = 1.0\ \textrm{cm}$

The magnetic field distribution calculated by Opera3D is illustrated in Fig. 3. Both the inner and outer coils are configured with a cross-section of $10 \times 10\ \textrm{cm}^2$. They are driven by an excitation of $\pm 6.3 \times 10^5\ \mathrm {A\cdot turns}$. This configuration yields an overall current density of $63\ \mathrm {A/mm}^2.$ This conservative setting provides a substantial engineering safety margin for future mechanical and structural optimizations.

Figure 3(a) illustrates the axial magnetic field $B_z$ on the mid-plane. The field magnitude reaches approximately 0.8 T in the guiding area and 2.46 T in the bending area. The simulation explicitly models the 1.1 m thick return yoke located between $r_L = 4.4$ m and 5.5 m. Within this region, the maximum magnetic flux density reaches *-2.35* T. This value approaches the ferromagnetic saturation limit of standard low-carbon steel but remains technically manageable. Furthermore, this realistic yoke geometry effectively shields the fringe field, keeping the magnetic leakage immediately outside the device ($r_L > 5.5$ m) below 10 Gauss. Figure 3(b) displays the radial component $B_r$ on the offset plane. These prominent $B_r$ features provide the primary edge-focusing forces for the non-bending plane. The azimuthal component $B_\theta$ is identically zero in the whole magnetic profile due to the model axisymmetry.

### Reference trajectories and injection conditions

**Fig. 4:**
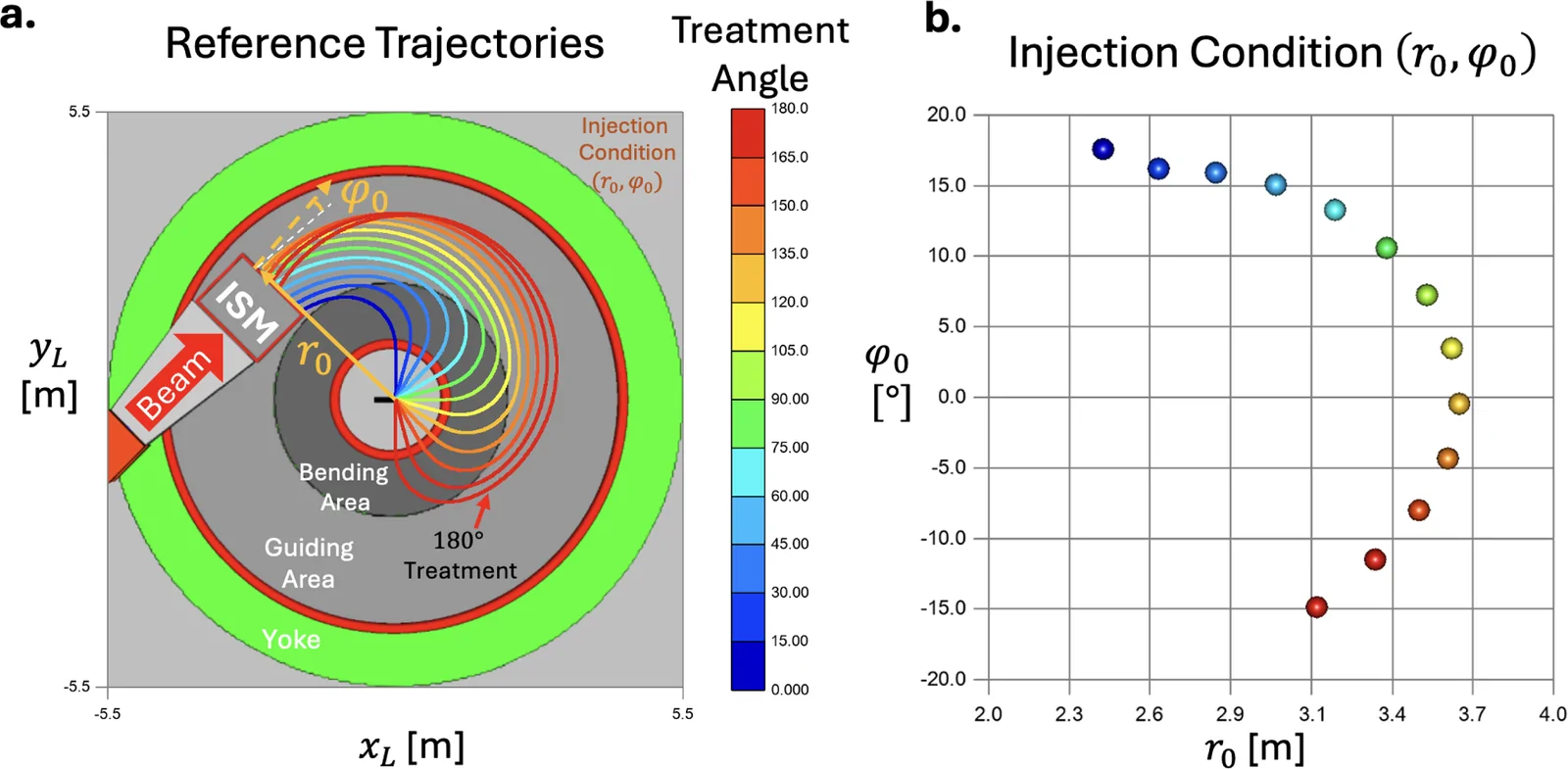
Reverse tracking results for beam injection. **a** Backtracked reference trajectories in the mid-plane for treatment angles from $0^\circ$ to $180^\circ$. For visual clarity, only trajectories at $15^\circ$ intervals are plotted. **b** The corresponding injection condition locus mapping the required radial position $r_0$ and entrance angle $\varphi _0$ at the ISM exit

Figure 4(a) displays representative reference trajectories. The reverse integration was executed in $1^\circ$ step across the entire $0^\circ$ to $180^\circ$ range. However, the plot illustrates trajectories every $15^\circ$ to maintain visual clarity. For every target angle the reverse calculation converges to a unique injection condition. Figure 4(b) maps these injection coordinates $(r_0, \varphi _0)$ for all plotted angles. These values determine the necessary upstream beam steering performed by the ISM.

### Beam transport evaluation and optical limits

Forward beam transport is evaluated by combining the backtracked injection conditions with the phase-space sampling strategy (Sect. 2.4.1). Propagating this conservative initial beam through the unoptimized 3D magnetic field provides an illustrative baseline of the system’s optical limits.

#### Detailed tracking analysis at $\theta _T=180^\circ$

**Fig. 5:**
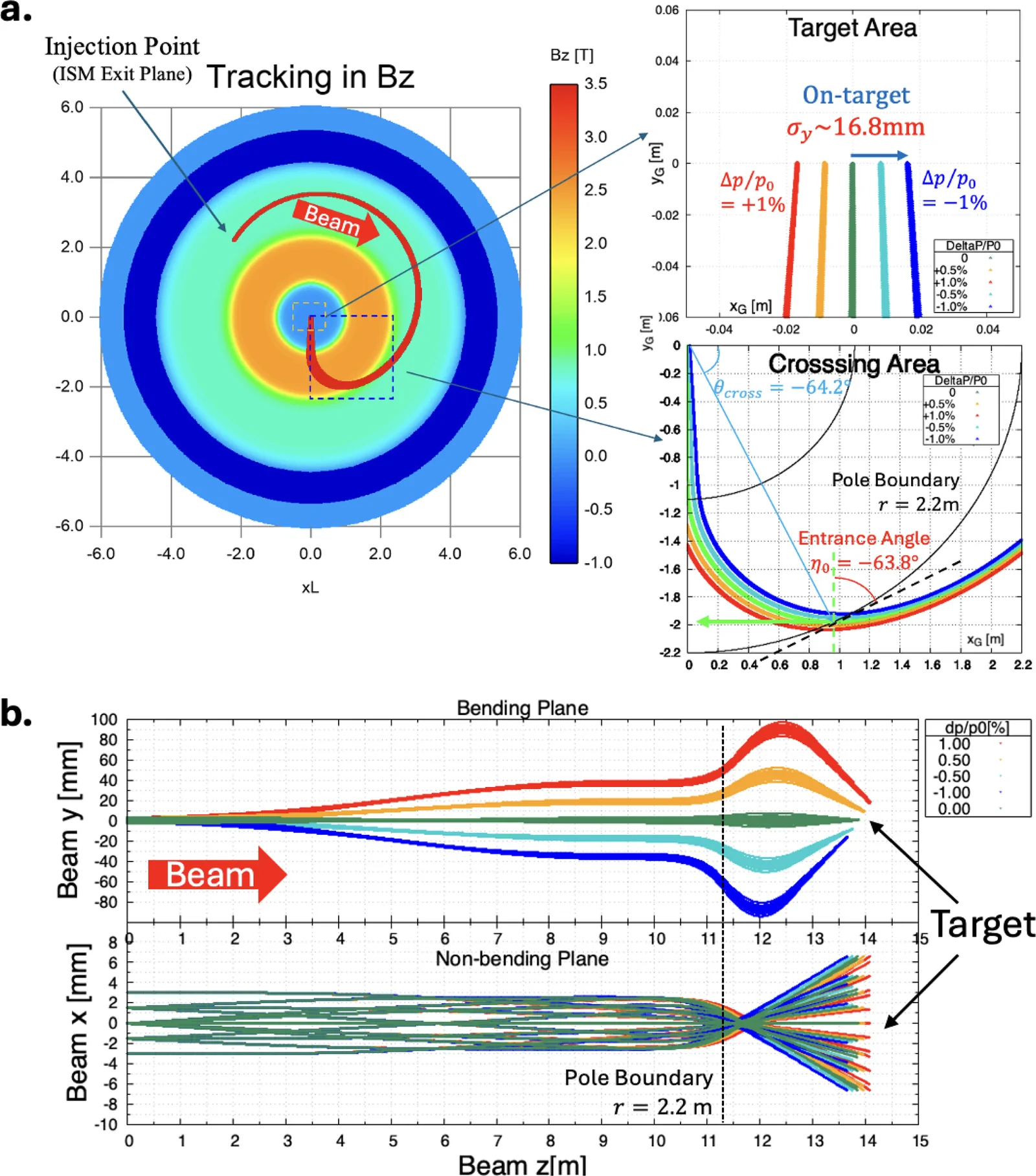
Illustrative example of forward beam ($\Delta p/p_{0}=\pm 1.0\%$) transport evaluation within the unoptimized baseline magnetic field for the $\theta _T=180^\circ$ treatment angle. **a** Laboratory-frame trajectory illustrating beam bending plane, overlaid on the mid-plane $B_z$ field map. Target and boundary crossing areas are magnified. **b** Beam-attached envelope evolution along the longitudinal coordinate $z_b$. The upper and lower panels correspond to the bending and non-bending planes, respectively. Colors denote momentum spread (red *+1%*, dark green 0, blue *-1%*), and the vertical dashed line indicates the field boundary at $r_L=2.2\ \textrm{m}$

To demonstrate the framework’s diagnostic capabilities, Fig. 5 isolates the distinct optical mechanisms driving beam divergence. In the bending plane, the simulation explicitly maps the onset of chromatic dispersion as the beam crosses the internal boundary with a large entrance angle ($\eta \sim 64^\circ$). Conversely, the non-bending plane reveals a decoupled transport mechanism. Because momentum trajectories here remain entirely overlapped, the visualization isolates the pronounced edge-focusing effect induced by the same boundary crossing. By explicitly capturing these contrasting behaviors (dispersion versus premature focusing), the 3D platform effectively confirms the need for plane-specific magnetic field shaping.

#### Continuous angular evaluation of on-target spot sizes

**Fig. 6:**
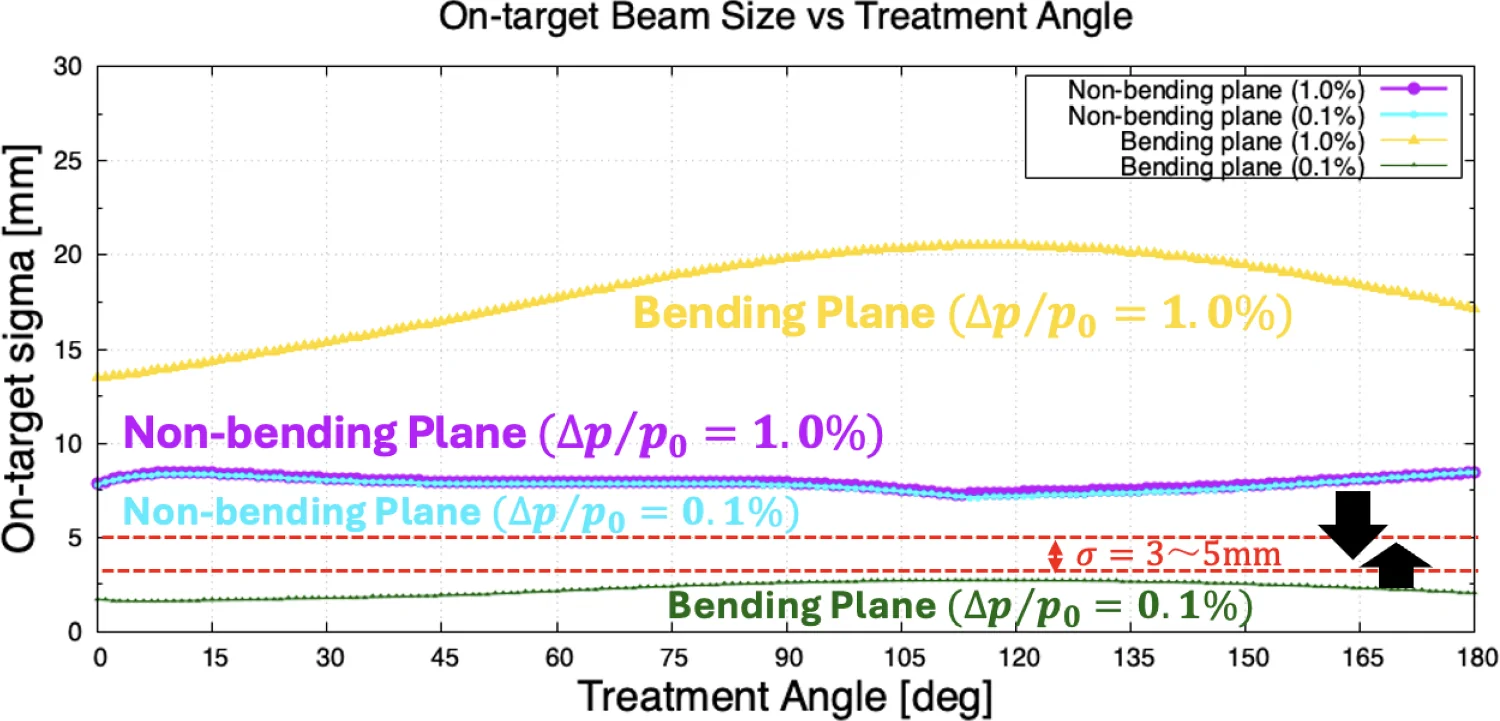
Example evaluation of on-target rms beam spot sizes within the unoptimized baseline field, calculated at 1-degree intervals across the continuous $0^\circ$ to $180^\circ$ treatment range. This continuous tracking capability intuitively visualizes both the angle dependence and the beam spot symmetry between the two transverse planes. Currently, the bending plane (golden for 1.0% and green for 0.1% momentum spread) is driven by chromatic dispersion, while the non-bending plane (magenta for 1.0% and cyan for 0.1%) is dominated by edge-focusing. The ultimate optimization goal is to shape the magnetic field such that both envelopes overlap within the clinically practical $\sigma =3\sim 5$ mm target band (red dashed lines), ensuring excellent beam spot symmetry

A key advantage of the 3D framework is its capability to generate comprehensive, high-resolution optical maps across the entire clinical range (Fig. 6). Rather than assessing isolated angles, this continuous evaluation directly visualizes the severe cross-plane asymmetry inherent to the unoptimized baseline design. The distinct responses to momentum spread reduction cleanly separate the governing optics: chromatic dispersion dictates the bending plane, while stable localized edge-focusing dictates the non-bending plane. Ultimately, this quantitative mapping serves as the exact benchmark for future design iterations: successful 3D magnetic field optimization will be achieved when both envelopes are steered to overlap within the clinically desirable target zone.

## Discussion

***Role of the 3D computational framework.*** Previous studies established the conceptual design of the static gantry using idealized 2D magnetic fields [8]. However, transitioning to the robust beam optic design stage requires evaluating 3D fringe fields and non-axisymmetric structures where analytical methods reach their inherent limits. Although the present baseline field remains axisymmetric, advanced pole-shaping strategies and decoupled ISM concepts are expected to introduce non-axisymmetric field components. To accurately evaluate the impact of complex fringe fields on beam optics and to enable the future modeling of these structures, we developed the present computational workflow. Additionally, this approach uniquely enables a closed-loop design: integrating 3D magnetic field modeling in Opera3D with custom phase-space tracking to iteratively extract trajectory-specific optics and feedback into the field geometry. Specifically, it may provide reconstructed on-target Twiss parameters and dispersion functions for beam optics optimization. Furthermore, the custom tracking execution typically completes within minutes, while the 3D field maps may be iteratively updated within hours, ensuring the computational efficiency required for systematic evaluations. Ultimately, this platform provides a practical foundation to explore and evaluate complex components from conceptual ideas into calculable robust beam optic designs.

***Beam transport performance.*** The evaluations highlight distinct optical behaviors for each transverse plane. In the bending plane, the conservative 1% momentum spread, which represents a scenario with an upstream degrader, drives pronounced chromatic dispersion. However, as explicitly demonstrated by our comparative tracking results, this dispersion is effectively mitigated by utilizing a downstream energy degrader. This concept assumes a placement directly in front of the patient, similar to a Mevion system [10, 11], within the field-free treatment area ($r_L < 1.0$ m). Although a detailed mechanical design covering all treatment angles requires further investigation, it may conceptually involve a stationary ring-shaped degrader [8] or an adaptive rotating nozzle. Assuming a typical momentum spread of 0.1% in a proton beam from a cyclotron [12], this approach successfully reduces the bending-plane spot size to practical levels. Conversely, the non-bending plane optics are dominated by boundary edge-focusing and initial Twiss parameters, rather than the sensitivity to momentum spread. Clinical beam emittance and practical spot sizes in modern pencil-beam scanning systems [12, 13] typically exceed the conservative values assumed in the present baseline evaluation. Therefore, the stable optical magnification observed in the non-bending plane will have a more severe impact on the absolute clinical spot size, making the mitigation of edge-focusing a primary target for future optimization.

***Future outlook: optical and structural optimization.*** Moving forward, our 3D computational framework will serve as a practical tool for comprehensive design optimization. With respect to the beam optics, the compact design prevents the use of conventional quadrupoles, meaning transverse focusing relies primarily on customized 3D magnetic field modulations. We will utilize this framework to evaluate potential solutions, such as introducing localized “hill and valley” field variations along the regional boundaries to provide robust, distributed edge-focusing. This specific strategy may help compress the factor-of-two beam size discrepancy observed in the unoptimized baseline. Preliminary studies indicate that this modulation provides sufficient optical degrees of freedom to achieve the required focusing strength, demonstrating that the current baseline is not fundamentally limited by simple edge focusing alone.

Structurally, the expanded 5.5 m radius noted in Sect. 2.1 was deliberately chosen to maximize spatial freedom for initial optics evaluations. A subsequent structural objective is to shrink this radial footprint and reduce overall weight. For instance, truncating unused yoke sections into an asymmetric “C-shape” will significantly lighten the device. This optimization facilitates easier clinical implementation, complementing the system’s exceptionally compact axial thickness (under 2 m).

Regarding beam injection, the current baseline routes the trajectory through the return yoke. We acknowledge that introducing a yoke gap may distort the internal magnetic field and gently influence beam propagation. Such optical distortions could potentially be mitigated by pre-compensating the beam utilizing upstream quadrupoles. To proactively address this structural challenge, the injection could be relocated to the outermost radius in subsequent design iterations. Furthermore, the present evaluation focuses on a $180^\circ$ continuous range. A realistic extension to approximately $200^\circ$ may further reduce the necessity of rotating the patient during treatments. This range already aligns with certain commercial solutions, where full target coverage can be achieved by reversing the patient bench. However, relocating the injection to the outermost radius could potentially avoid geometric interference entirely. This structural evolution might enable rapid, continuous $360^\circ$ irradiation from a single beamline.

## Conclusion

In this work, we established a 3D computational framework integrating Opera3D magnetic field modeling with phase-space tracking. Utilizing this tool, we numerically evaluated the baseline beam transport of the static gantry and identified its primary optical limitations. Specifically, our evaluations under typical momentum-spread conditions suggested that reducing the momentum spread substantially mitigates bending-plane divergence, whereas the non-bending plane remains highly constrained by boundary edge-focusing and initial Twiss parameters.

Moving forward, the developed platform will support future iterative field optimization, such as customized pole-shaping to mitigate edge-focusing, and the evaluation of non-axisymmetric structures in the beam injection line. Overall, this framework provides a practical foundation to advance the static gantry concept toward a robust beam optic design.

## Data Availability

The datasets and simulation inputs are available from the corresponding author on reasonable request.

## Declarations

### Use of AI-assisted tools

The authors used a large language model only for English language polishing of author-written text. No results or figures are generated by AI. All scientific content is conceived, validated, and is fully accountable by the authors. No generative AI images are used.
